# Antibacterial activity of standard and N-doped titanium dioxide-coated endotracheal tubes: an *in vitro* study

**DOI:** 10.5935/0103-507X.20170009

**Published:** 2017

**Authors:** Valentina Caratto, Lorenzo Ball, Elisa Sanguineti, Angelo Insorsi, Iacopo Firpo, Stefano Alberti, Maurizio Ferretti, Paolo Pelosi

**Affiliations:** 1 Dipartimento di Chimica e Chimica Industriale, Università degli Studi di Genova, Genova, Italy.; 2 Istituto CNR SPIN - Genova, Italy.; 3 Dipartimento di Scienze Chirurgiche e Diagnostiche Integrate, Università degli Studi di Genova, Genova, Italy.; 4 Dipartimento di Scienze della Terra, dell'Ambiente e della Vita, Università degli Studi di Genova, Genova, Italy.

**Keywords:** Pneumonia, ventilator associated, Intubation, intratracheal/instrumentation, Titanium, Coated materials, biocompatible, Antibacterial agents

## Abstract

**Objective:**

The aim of this study was to assess the antibacterial activity against *Staphylococcus aureus* and *Pseudomonas aeruginosa* of two nanoparticle endotracheal tube coatings with visible light-induced photocatalysis.

**Methods:**

Two types of titanium dioxide nanoparticles were tested: standard anatase (TiO_2_) and N-doped TiO_2_ (N-TiO_2_). Nanoparticles were placed on the internal surface of a segment of commercial endotracheal tubes, which were loaded on a cellulose acetate filter; control endotracheal tubes were left without a nanoparticle coating. A bacterial inoculum of 150 colony forming units was placed in the endotracheal tubes and then exposed to a fluorescent light source (3700 lux, 300-700 nm wavelength) for 5, 10, 20, 40, 60 and 80 minutes. Colony forming units were counted after 24 hours of incubation at 37°C. Bacterial inactivation was calculated as the percentage reduction of bacterial growth compared to endotracheal tubes not exposed to light.

**Results:**

In the absence of light, no relevant antibacterial activity was shown against neither strain. For *P. aeruginosa*, both coatings had a higher bacterial inactivation than controls at any time point (p < 0.001), and no difference was observed between TiO_2_ and N-TiO_2_. For *S. aureus*, inactivation was higher than for controls starting at 5 minutes for N-TiO_2_ (p = 0.018) and 10 minutes for TiO_2_ (p = 0.014); inactivation with N-TiO_2_ was higher than that with TiO_2_ at 20 minutes (p < 0.001), 40 minutes (p < 0.001) and 60 minutes (p < 0.001).

**Conclusions:**

Nanosized commercial and N-doped TiO_2_ inhibit bacterial growth under visible fluorescent light. N-TiO_2_ has higher antibacterial activity against *S. aureus* compared to TiO_2_.

## Abstract (pt)

**Objetivo:**

Avaliar a atividade antibacteriana contra *Staphylococcus aureus* e *Pseudomonas* aeruginosa de dois revestimentos endotraqueais com nanopartículas e fotocatálise sob luz visível.

**Métodos:**

Testaram-se dois tipos de nanopartículas de titânio: anatase padrão (TiO_2_) e TiO_2_ nano-dopada (N-TiO_2_). As nanopartículas foram colocadas em superfície interna de segmentos de tubos endotraqueais comerciais, aplicadas sobre um filtro de acetato de celulose; os tubos endotraqueais controle foram deixados sem revestimento de nanopartículas. Em cada tubo endotraqueal foi inoculado um total de 150 unidades formadoras de colônia e, a seguir, estes foram expostos a uma fonte de luz fluorescente (3700 lux, comprimento de onda de 300 - 700nm) por 5, 10, 20, 40, 60 e 80 minutos. Contaram-se as Unidades Formadoras de Colônia após 24 horas de incubação a 37ºC. A inativação bacteriana foi calculada como a redução porcentual do crescimento bacteriano em comparação a tubos não expostos à luz.

**Resultados:**

Na ausência de luz, não se observou qualquer atividade antibacteriana relevante contra qualquer das cepas estudadas. Para *P. aeruginosa*, ambos os revestimentos tiveram inativação bacteriana mais elevada do que o controle em qualquer dos momentos de avaliação (p < 0,001), sendo que não se observaram diferenças entre o revestimento padrão e nano-dopado. Para *S. aureus*, a inativação foi maior que os controles, começando a partir de 5 minutos para nano-dopado (p = 0,018) e 10 minutos para o revestimento padrão (p = 0,014); a inativação com a forma nano-dopada foi maior do que com a forma padrão aos 20 minutos (p < 0,001), 40 minutos (p < 0,001) e 60 minutos (p < 0,001).

**Conclusões:**

O revestimento com nanopartículas de titânio comercial padrão e nano-dopado inibiu o crescimento bacteriano sob a luz fluorescente visível. o revestimento nano-dopado teve maior atividade antibacteriana contra *S. aureus* em comparação à atividade observada com o revestimento com anatase padrão.

## INTRODUCTION

Patients undergoing mechanical ventilation in the intensive care unit (ICU) are at risk of several complications, including ventilator-associated pneumonia (VAP).^(1)^ Despite heterogeneity in its definition,^(2)^ VAP is a disease that may affect as many as a quarter of all mechanically ventilated patients^(3)^ and has the potential to double the risk of mortality and increase the costs of care and the duration of hospitalization and mechanical ventilation.^(4)^ New guidelines and a bundle of simple interventions are being developed to identify VAP and to reduce its incidence.^(5-7)^

One of the factors contributing to the pathogenesis of VAP is believed to be the rapid colonization of biofilm-forming pathogens, such as *Pseudomonas aeruginosa* and *Staphylococcus aureus,* on the surface of endotracheal tubes (ETT).^(8)^ These biofilms constitute a protective environment for bacterial colonies, also possibly contributing to the development of resistance to antibacterial drugs.^(9)^

Several antibacterial internal coatings have been proposed to reduce ETT bacterial colonization, including silver sulfadiazine and chlorhexidine,^(10)^ antibiotics^(11)^ and nanoparticle-sized titanium dioxide (TiO_2_) alone or with silver.^(12)^ Photocatalysis was proposed as an additional method to further increase the antimicrobial activity of coatings,^(12,13)^ but it has not been employed because the catalysts investigated in the first studies required an ultraviolet (UV) light source at the bedside to activate the antibacterial effect. Photocatalysis is more effective on gram-negative than gram-positive bacteria due to differences in cell wall composition.^(14)^

Recently, modified forms of nanoparticle-sized TiO_2_ are under investigation due to their capability of showing photocatalytic antibacterial activity in the spectrum of visible light,^(15-17)^ thus avoiding the use of potentially harmful UV light and obtaining a bactericidal effect with conventional fluorescent light illumination,^(18)^ widely available in hospital environments. TiO_2_ exhibits its catalytic activity when irradiated with ultraviolet and visible light, while the action of other catalysts, such as Cu^(19,20)^ or Ag^(21)^ nanoparticles, is mainly explained by their chemical composition due to the release of metal ions in the suspension medium. In fact, for metal nanoparticles, it is crucial to control size, shape, composition, chemical and physical properties during the synthesis process; therefore, TiO_2_ was used for its cost-effectiveness^(22,23)^ and its easy synthetic process, which appears to be much easier than the techniques used to synthesize metal nanoparticles, such as sacrificial anode electrolysis or thermal plasma techniques.

The aim of this study was to assess in vitro the efficacy of two forms of TiO_2_ as internal coating agents of ETTs segments by examining their ability to inhibit the growth of two bacteria commonly involved in VAP, *S. aureus* and *P. aeruginosa*, through photocatalysis under visible light. We hypothesized that N-doping of TiO_2_ could potentiate the antibacterial effect, also allowing photocatalysis under visible light, especially in gram-positive bacteria, where photocatalysis is less efficient.

## METHODS

In this study, two different forms of TiO_2_ nanoparticles were used: commercially available anatase (Sigma Aldrich, USA) and self-produced N-doped TiO_2_ (N-TiO_2_) crystallized in the anatase structure. N-TiO_2_ nanoparticles were synthesized using the sol-gel method, as described in a previous study.^(24)^ Briefly, 37.5mL titanium isopropoxide (Sigma-Aldrich) with 70mL of 2-propanol (Sigma-Aldrich) and 9mL aqueous solution of NH_3_ (15% V/V) were stirred at room temperature for 4 hours, then washed with deionized water and dried at 105°C for 12 hours. The xerogel was finally crushed into a fine powder and calcined at 350°C for 1 hour to complete the crystallization process.

Diffuse reflectance spectrophotometry (JASCO V-570 UV-VIS-NIR, Jasco Int. Co. Ltd., Japan), Brunauer-Emmett-Teller (BET, ASAP 2000, Micromeritics, US), X-ray powder diffraction (XRPD, Philips PW 1830 generator, 40 kV, 30 mA) and transmission electron microscopy (TEM, JEOL JEM 2010, lanthanum boride crystal operated at 200 kV) were used to characterize the nanoparticles. Scanning electron microscopy (SEM) with energy-dispersive X-ray spectroscopy (EDS) (StereoScan 360 microscope, Leica Cambridge Instruments, United Kingdom) was used to study the nanoparticles and the samples. EDS analysis was performed on different areas of a representative sample to estimate the chemical composition corresponding to each observed morphological pattern.

### Preparation of endotracheal tube segments and bacterial inoculation

Titanium dioxide was placed on the internal surface of a 5cm segment of an 8.0mm polyvinyl chloride commercial ETT (Rüsch, Kernen, Germany) loaded on a cellulose acetate filter with a 0.45µm pore size (Sartorius Stedim Biotech, Gottingen, Germany) at a concentration of 0.052mg/cm^2^ as previously described.^(17,25)^

Bacterial cultures were grown in *Luria Bertani* broth medium at 37°C, harvested at the exponential growth phase, washed and suspended in 0.9% sterile sodium chloride solution to a final colony forming unit (CFU) count of 30 ± 5 per milliliter, as verified by optical density at 600nm (Eppendorf AG, Hamburg, Germany). Five-milliliter aliquots of such bacterial suspension were filtered through the cellulose acetate filter or the TiO_2_-treated cellulose acetate filter to depose the CFUs. The inoculum size we used had a similar CFU count compared to a previous study^(17)^ and has been shown to be able to initiate biofilm formation *in vitro*.^(26)^ The filter was later inserted in the ETT segment. A small bacterial inoculum was chosen to simulate the initial colonization of the tube, subsequently leading to the formation of biofilm. Sample ETTs were internally coated with TiO_2_ or N-TiO_2_ on cellulose acetate, and controls were only coated with cellulose acetate.

### Photocatalytic activity

**Figure 1:**
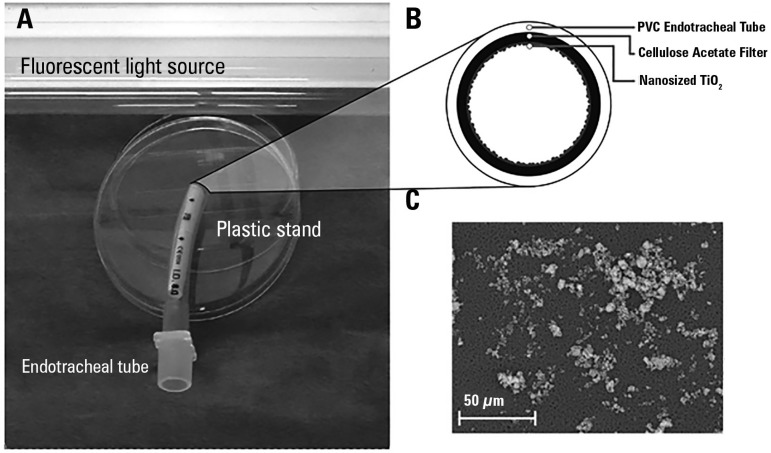
Experimental setting (panel A), tube coating scheme (panel B) and nanoparticle deposition on acetate filter (panel C).

Sample and control ETTs were exposed to fluorescent light generated by a commercial fluorescent light source, a neon tube with 300 - 700nm wavelength emission spectrum and 3700 lux illuminance (Neon L36W/640, Osram, Münich, Germany) placed 15cm from the ETT outlet for different exposure times: 5, 10, 20, 40, 60 and 80 minutes. Figure 1 illustrates the experimental setup and the nanoparticle deposition on acetate filter.

At the end of each exposure time, acetate filters with bacterial colonies were removed from the ETT and placed at 37°C on appropriate selective agar media: MacConkey agar for *P. aeruginosa* and mannitol-salt agar for *S. aureus*. After 24 hours of incubation at 37°C, CFUs were counted.

Blank ETT segments were coated with cellulose acetate without TiO_2_, placed in a dark room after bacterial inoculation, and analyzed at the same time points as the sample and control tubes: they were used as references to calculate contingent bacterial inactivation not attributable to the photocatalysis processes.

The percentage of inactivation (I) due to the photocatalytic activity was calculated, as previously described,^(17)^ as the percent reduction of CFUs in the samples and control tubes compared to the blank ETTs, with the formula I(%) = (N_B_-N)/N_B_ *·* 100, where N_B_ is the number of CFUs on the filter without TiO_2_ and without light exposure (blank), and N is the number of CFUs on examined nanoparticle-treated (sample) or untreated (control) ETTs.

### Statistical analysis

The kinetics of bacterial inactivation were calculated from the data of three independent repeated measurements. Data were compared by two-way analysis of variance (ANOVA) with Tukey's post hoc test, and statistical significance was considered at p < 0.05. Data were reported as mean ± standard deviation or as the percentage of inactivation if not otherwise stated. Experimental data were analyzed using Statistical Package for the Social Science (SPSS) version 21 (IBM Corp, USA).

## RESULTS

### TiO**_2_** nanoparticle characterization

Diffuse reflectance analysis showed wavelength absorption values of 384nm and 413nm for TiO_2_ and N-TiO_2_, respectively, thus representing a shift of the absorption edge towards the visible region for the N-TiO_2_. BET analysis revealed specific surface area values of 120m^2^g^-1^ and 96.8m^2^g^-1^ for TiO_2_ and N-TiO_2_ nanoparticles, respectively. XRPD and TEM analyses revealed that both the commercial TiO_2_ and N-TiO_2_ crystal structure contained anatase, with a particle size of 19 ± 2nm and 17 ± 2nm, respectively.

### Bacterial inactivation kinetics

In the absence of light, no relevant antibacterial activity was shown against both strains, with bacterial inactivation after 80 minutes remaining below 3.5% for both TiO_2_ and N-TiO_2_.

**Figure 2:**
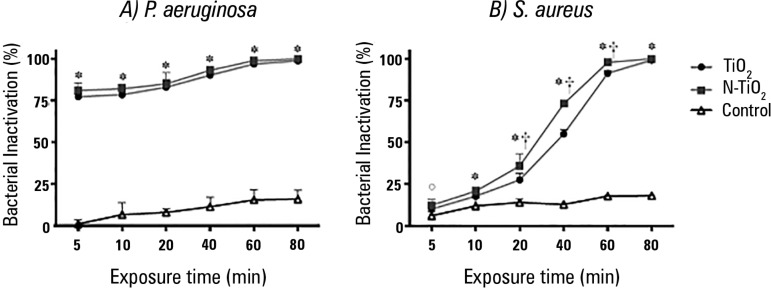
Inactivation kinetics of *Pseudomonas aeruginosa* (A) and *Staphylococcus aureus* (B) under fluorescent light. * The inactivation of TiO_2_ and N-TiO_2_ was significantly higher than that in controls (p < 0.05). ° Only N-TiO_2_ was more active than controls (p < 0.05). † N-TiO_2_ more active than TiO_2_ (p < 0.05).

The inactivation kinetics of the two bacterial strains under fluorescent light irradiation are illustrated in figure 2. In EETs exposed to irradiation without TiO_2_ (controls), bacterial inhibition ranging from 1% to 18% was found due to the dehydration induced by irradiation.

For *P. aeruginosa,* all TiO_2_-coated ETTs showed higher bacterial inactivation when compared to controls (p < 0.001 at all time points); no significant difference was found between commercial TiO_2_ and N-TiO_2_ (p > 0.20 at all time points).

For *S. aureus*, inactivation was higher than for controls starting at 5 minutes for N-TiO_2_ (p = 0.018) and 10 minutes for TiO_2_ (p = 0.014). For exposure times longer than 10 minutes, both coatings were more efficient than controls (p < 0.001 at each time point). N-TiO_2_ showed an inactivation higher than that for TiO_2_ at 20 minutes (p < 0.001), 40 minutes (p < 0.001) and 60 minutes (p < 0.001) of light exposure.

**Figure 3:**
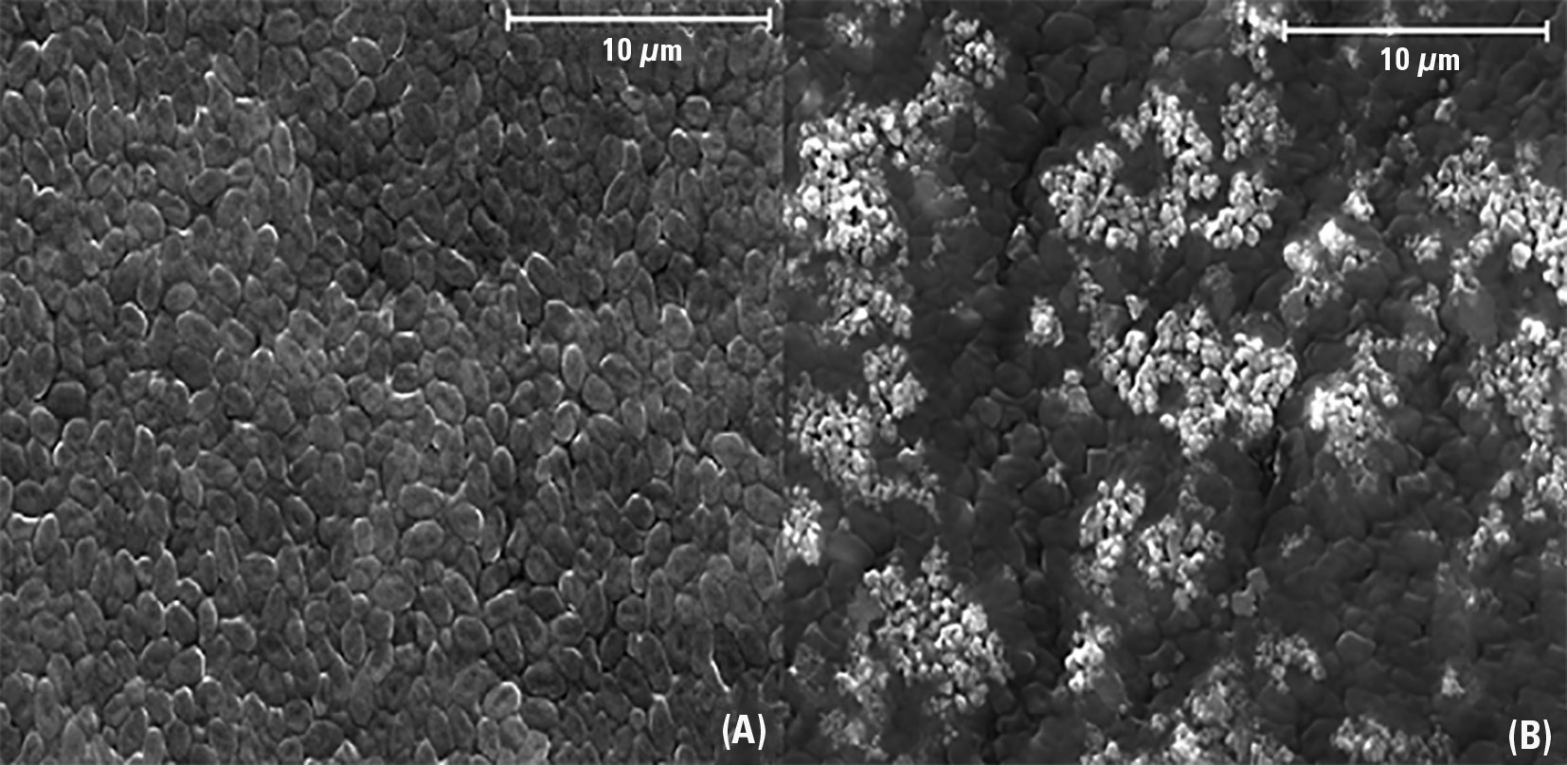
Scanning electron microscopy images (magnification: x 4000; extra high tension: 20 kV) of untreated Pseudomonas aeruginosa cells (A) and treated *Pseudomonas aeruginosa* cells (B) upon fluorescent light illumination in the presence of commercial TiO_2_ for 10 minutes.

**Figure 4:**
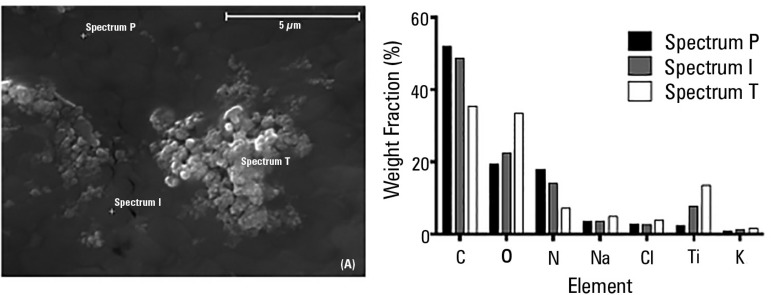
Scanning electron microscopy images of a representative sample of endotracheal tubes inoculated with *Pseudomonas aeruginosa* after 10 minutes of fluorescent light irradiation (left panel). P indicates an area where only the bacterial layer is visible, T represents an area where only TiO_2_ is visible, and I represents an intermediate region. The right panel shows element weight fractions (%) according to the energy-dispersive X-ray spectroscopy spectra analysis of a representative sample of *Pseudomonas aeruginosa* after 10 minutes of treatment with TiO_2_ under fluorescent light irradiation.

Figure 3 shows representative SEM images of *P. aeruginosa* deposed on the internal surface of an ETT coated with commercial TiO_2_ before (a) and after (b) 10 minutes of fluorescent light irradiation. Figure 4 shows a representative sample of N-TiO_2_-loaded ETT inoculated with *P. aeruginosa* after 10 minutes of light exposure with semi-quantitative EDS spectral analysis.

## DISCUSSION

The main findings of this study are the following: 1) in the absence of light, neither N-TiO_2_ nor TiO_2_ could inhibit bacterial growth within 80 minutes; 2) photocatalysis under fluorescent visible light inhibited bacterial growth in ETTs; 3) no difference in bacterial inactivation efficacy was observed between N-TiO_2_ and TiO_2_ for *P. aeruginosa*; and 4) bacterial inactivation was higher in N-TiO_2_-coated tubes compared to TiO_2_ for *S. aureus*.

This is the first study to investigate a potential clinical application of N-TiO_2_. Differently from previous studies, a small CFU count was deposed in the inoculum, mimicking the initial tube contamination that might occur *in vivo*. Absorbance measurements confirmed a shift of the absorption edge towards the visible region in N-TiO_2_ compared to commercial TiO_2_ due to a decrease of the energy gap of the lattice, which explains the better efficiency of N-TiO_2_ found in the present study.

### Bacterial inactivation mechanism

Both TiO_2_ and N-TiO_2_ were anatase, as previously reported elsewhere,^(17,24)^ and in presence of light uncoated control showed a minimal inactivation activity, due to dehydration.^(25)^ As illustrated in figure 3, before irradiation (left panel), the TiO_2_ was not visible and was entirely covered by a bacterial mat. After exposure to visible light (right panel), the white areas corresponding to the underlying TiO_2_ were visible and surrounded by bacterial debris as well as intact cells. Upon EDS analysis, the presence of Na and Cl was mainly due to the solution in which bacteria were suspended (0.9% NaCl) and appeared homogeneously distributed in the three regions, whereas C and N were attributable to bacterial cell components (carbohydrates and proteins), and Ti was the photocatalyst. Oxygen is attributable both to organic components of the bacterial cell and TiO_2_. In the EDS spectrum of region P, the percentage of Ti was low because TiO_2_ was covered by a bacterial mat, while the spectrum of region I showed an increase of the photocatalyst signal probably due to damage to the bacterial cell wall, reflected by a symmetrical increase in intracellular Na and K content. Finally, the spectrum of region T, corresponding to an area in which TiO_2_ was visible, indicates a high percentage of Ti, Na, K and a low content of C and N.

These findings suggest that the direct contact between bacteria and TiO_2_ on the filter surface increased the extent of oxidative damage, enhancing killing of bacteria in a short time according to mechanism proposed by Foster et al.^(27)^ The authors suggested a mechanism involving initial damage in the contact areas between the cells and TiO_2_, affecting membrane permeability, followed by increased damage to all cell wall layers, allowing leakage of small molecules such as ions. Further membrane damage allows the leakage of higher molecular weight components, such as proteins. This may be followed by protrusion of the cytoplasmic membrane into the surrounding medium through degraded areas of the peptidoglycan and, eventually, lysis of the cell. Degradation of the internal components of the cell then occurs, followed by complete mineralization.

The advantages of N-TiO_2_ compared to TiO_2_ in terms of bacterial inactivation were not explained by different crystalline forms, as both nanoparticles were made of anatase with comparable sizes. The slight difference between the BET values indicated that the different performances of the two nanoparticles might then be explained by the lower value of the energy gap, which allows major efficiency in the visible photon absorption and in the photocatalytic antimicrobial activity. It must also be noticed that the presence of further defects, such as doping atoms, in the TiO_2_ lattice enhances electron trapping, which is one of the main mechanisms involved in photocatalytically activated processes.

### Bacterial inactivation kinetics

In the absence of fluorescent light, bacteria impregnated on the internal surface of endotracheal tubes were insensitive to the two nanoparticle coatings in the examined time window.

In the present study, both TiO_2_ and N-TiO_2_ demonstrated the inactivation of *P. aeruginosa* growth higher than 77% after 5 minutes of exposure (Figure 2). The bactericidal activity began within 5 or 10 minutes depending on the strain and coating and increased with a longer irradiation time. The photocatalytic inactivation appears to be similar to that obtained in a previous study on *Escherichia coli*.^(17)^

In ETTs inoculated with *S. aureus*, bacterial inactivation rates comparable to those obtained for *P. aeruginosa* were achieved after 40 minutes and 60 minutes in samples treated with N-TiO_2_ and TiO_2_, respectively. As shown in figure 2, after 5 minutes of light exposure, there were no significant differences between TiO_2_ and control ETTs. After 20, 40 and 60 min, N-TiO_2_ was more active than TiO_2_, and for longer exposure times (80 min), the photocatalytic inactivation reached a plateau at nearly 100% (99 ± 1)%. Previous studies evaluated commercial TiO_2_ without light exposure supplementation and found a lack of activity against *S. aureus*.^(12)^

The kinetics of the photocatalytic inactivation of *P. aeruginosa* were equal for TiO_2_ and N-TiO_2_ and faster than those of *S*. aureus, in accordance with data found by other authors who observed that gram-positive bacteria are more resistant to photocatalysis than gram-negative bacteria.^(12,27,28)^

### Clinical implications

According to our findings, fluorescent light irradiation may be sufficient to trigger the photocatalytic activity of TiO_2_ and N-TiO_2_ without the need for an ultraviolet light source. N-TiO_2_ shows a higher and faster bacterial inactivation than TiO_2_ when tested against *S. aureus*. Our results suggest that photocatalysis under fluorescent visible light, especially with N-TiO_2_, could have clinical applications.

These results showed that nanosized N-TiO_2_ can effectively prevent the growth of a small bacterial inoculum when exposed to conventional fluorescent light: this finding could have several practical applications. Nanoparticles-coated materials could be used to prevent or reduce bacterial colonization of medical devices as well as furniture and working surfaces, especially in environments like the ICU, where the high prevalence of multi-resistant strains of bacteria requires non-pharmaceutical measures to control the spread of infection.

It is therefore important to optimize the chemical, physical and morphological properties of this material to further improve disinfection under visible light and study the activity of other microorganisms. In this bench-top study, nanoparticles were loaded in the ETT deposed on an acetate filter. For clinical applications, the development of a method to depose the nanoparticles on the polymeric matrix of the tube is necessary.

### Limitations of the study

A major limitation of this study was that a single light source was tested: we are therefore unable to estimate a threshold of light irradiation necessary to initiate the photocatalytic process. Since ETTs are placed deeply inside the trachea, further studies should investigate whether ambient light transmission through the tube wall is sufficient to activate photocatalysis or whether an especially designed light source should be placed nearby to allow the antimicrobial activity. In the latter case, the fact that N-TiO_2_ is more effectively activated by fluorescent light without the need for a UV source would increase the clinical feasibility of such an approach. The efficacy of the coating was not tested against an active comparator, such as an antibiotic-loaded coating. However, the emergence of multi-drug resistant strains of bacteria in the ICU encourages research in infection control strategies not implying the use of antibiotics. Moreover, further studies are warranted to investigate the antibacterial activity of N-TiO_2_ against other bacterial strains and its applicability *in vivo*.

## CONCLUSIONS

The photocatalytic inactivation of *P. aeruginosa* and *S. aureus* by commercial TiO_2_ and synthesized N-TiO_2_ was analyzed. Nanosized commercial TiO_2_ and N-TiO_2_ inhibit bacterial growth under visible fluorescent light. N-TiO_2_ has higher antibacterial activity against *S. aureus* compared to TiO_2_. The results of this study show that photocatalysis with the N-TiO*_2_* method was an effective tool for bacteria inactivation. According to our findings, natural light irradiation or room light could be sufficient for N-TiO_2_ activation, so this disinfection method could be constantly operating.

### Authors' contributions

V Caratto and L Ball equally contributed to this manuscript. V Caratto, L Ball and E Sanguineti designed the study, collected the data, and wrote and revised the manuscript. M Ferretti and P Pelosi designed the study, interpreted the results and wrote and revised the manuscript. I Firpo and S Alberti collected the data and revised the manuscript. L Ball and A Insorsi performed data analysis and revised the manuscript. All the authors revised the final version of the manuscript.
